# Spatially Discordant Alternans and Arrhythmias in Tachypacing-Induced Cardiac Myopathy in Transgenic LQT1 Rabbits: The Importance of I_Ks_ and Ca^2+^ Cycling

**DOI:** 10.1371/journal.pone.0122754

**Published:** 2015-05-13

**Authors:** Emily Lau, Konstantinos Kossidas, Tae Yun Kim, Yukiko Kunitomo, Ohad Ziv, Song Zhen, Chantel Taylor, Lorraine Schofield, Joe Yammine, Gongxin Liu, Xuwen Peng, Zhilin Qu, Gideon Koren, Bum-Rak Choi

**Affiliations:** 1 Cardiovascular Research Center, Division of Cardiology, Rhode Island Hospital, Alpert Medical School of Brown University, Providence, Rhode Island, United States of America; 2 Department of Comparative Medicine, Pennsylvania State University College of Medicine, Hershey, Pennsylvania, United States of America; 3 Department of Medicine (Cardiology), David Geffen School of Medicine, University of California Los Angeles, Los Angeles, California, United States of America; University of Minnesota, UNITED STATES

## Abstract

**Background:**

Remodeling of cardiac repolarizing currents, such as the downregulation of slowly activating K^+^ channels (I_Ks_), could underlie ventricular fibrillation (VF) in heart failure (HF). We evaluated the role of *I*_ks_ remodeling in VF susceptibility using a tachypacing HF model of transgenic rabbits with Long QT Type 1 (LQT1) syndrome.

**Methods and Results:**

LQT1 and littermate control (LMC) rabbits underwent three weeks of tachypacing to induce cardiac myopathy (TICM). *In vivo* telemetry demonstrated steepening of the QT/RR slope in LQT1 with TICM (LQT1-TICM; pre: 0.26±0.04, post: 0.52±0.01, P<0.05). *In vivo* electrophysiology showed that LQT1-TICM had higher incidence of VF than LMC-TICM (6 of 11 vs. 3 of 11, respectively). Optical mapping revealed larger APD dispersion (16±4 vs. 38±6 ms, p<0.05) and steep APD restitution in LQT1-TICM compared to LQT1-sham (0.53±0.12 vs. 1.17±0.13, p<0.05). LQT1-TICM developed spatially discordant alternans (DA), which caused conduction block and higher-frequency VF (15±1 Hz in LQT1-TICM vs. 13±1 Hz in LMC-TICM, p<0.05). Ca^2+^ DA was highly dynamic and preceded voltage DA in LQT1-TICM. Ryanodine abolished DA in 5 out of 8 LQT1-TICM rabbits, demonstrating the importance of Ca^2+^ in complex DA formation. Computer simulations suggested that HF remodeling caused Ca^2+^-driven alternans, which was further potentiated in LQT1-TICM due to the lack of I_Ks_.

**Conclusions:**

Compared with LMC-TICM, LQT1-TICM rabbits exhibit steepened APD restitution and complex DA modulated by Ca^2+^. Our results strongly support the contention that the downregulation of I_Ks_ in HF increases Ca^2+^ dependent alternans and thereby the risk of VF.

## Introduction

Ventricular arrhythmia is a significant cause of mortality in heart failure (HF) patients [1–3]. Electrical and structural remodeling associated with HF have been proposed to increase vulnerability to ventricular fibrillation (VF) [2–4]. The hallmark of electrical remodeling in HF is a decrease in repolarization reserve that prolongs action potential duration (APD) [5–7], which is thought to promote triggered activity such as early and delayed afterdepolarizations and APD alternans, thereby enhancing reentry formation [8, 9].

Voltage-dependent K^+^ channels are critical in cardiac repolarization, and their downregulation is thought to play a major role in HF-related APD prolongation. The most consistently downregulated K^+^ channels in HF are the transient outward potassium current (I_to_) [10] and slowly activating delayed rectifier potassium current (I_Ks_) [6, 11, 12]. Since I_to_ rapidly inactivates during the plateau phase of action potentials, it is thought to have minimal impact on APD in large mammals [13, 14]. In contrast, I_Ks_ can play an essential role as repolarization reserve [15–18] in action potential repolarization when other repolarization currents are reduced [19–21]. Besides downregulation of repolarizing currents such as I_to_ and I_K1_, several depolarizing currents are upregulated, including late Na^+^ current [22–24] and Na^+^/Ca^2+^ exchanger current (I_NCX_) [25–28]. Hence, I_Ks_ downregulation in HF in conjunction with other ion channel remodeling may further accentuate APD prolongation and promote arrhythmogenesis.

Alternatively, I_Ks_ downregulation in HF may not necessarily be arrhythmogenic. Due to slow deactivation [29], the amplitude of I_Ks_ becomes larger during fast heart rates [19, 20, 30, 31]. As a result, I_Ks_ contributes APD shortening at short diastolic intervals to form the characteristic APD restitution curve [32]. Steep APD restitution has been linked to susceptibility to APD alternans [33–36], and the blockade of I_Ks_ may be effective against repolarization shortening in the setting of fast heart rate and flattened APD restitution, thereby protecting against reentry formation [37, 38]. In addition, due to its slow deactivation, I_Ks_ can promote post-repolarization refractoriness and enhance wavebreaks in VF [39]. Therefore, it is possible that the effect of I_Ks_ in HF remodeling may be compensatory and beneficial by preventing repolarization shortening and post-repolarization refractoriness. Overall, the reduction of I_Ks_ in HF can have either a pro-arrhythmic or an anti-arrhythmic effect depending on its amplitude and relative contribution to repolarization and restitution. Intracellular Ca^2+^ could also play a role in modulating I_Ks_ current density, as higher levels of intracellular Ca^2+^ increase I_Ks_ current [40].

Here we investigated the role of I_Ks_ in HF-related arrhythmias by inducing HF by a tachypacing protocol [41] in a transgenic rabbit model of LQT1 [42] and comparing that to HF induced in littermate control rabbits (LMC). We then compared susceptibility to developing alternans and VF induction in LQT1 rabbits that completely lack I_Ks_ vs. their LMCs in which tachypacing induced ~55% downregulation of I_Ks_ [43]. We found that total lack of I_Ks_ significantly increases arrhythmogenesis by increasing APD dispersion and promoting spatially discordant alternans (DA). This finding emphasizes the importance of I_Ks_ remodeling in promoting arrhythmogenesis in failing hearts.

## Methods

All animal experiments were performed in accordance with the local guidelines of the institutions and only after approval by the Institutional Animal Care and Use Committee (IACUC) at Rhode Island Hospital, in accordance with the Institute for Laboratory Animal Research (ILAR) Guidelines for the Care and Use of Laboratory Animals published by the US National Institutes of Health (NIH publication #85–23; Revised 1996). Adult male New Zealand white rabbits were selected from LMC and LQT1 lines and age-matched.

### Tachypacing-induced Cardiomyopathy (TICM) Protocol

Tachypacing-induced cardiomyopathy [41] was used to model dilated, non-ischemic cardiomyopathy. A programmable right ventricular (RV) pacemaker (Medtronic) was implanted in adult male LQT1 and LMC rabbits in a sterile surgical suite. Rabbits were anesthetized with IM ketamine/xylazine (25 mg/kg; 3.75 mg/kg body weight) and buprenorphine (0.03 mg/kg subcutaneously), followed by intubation and ventilation with inhaled isoflurane (1–2%, FiO2 0.5). A neck dissection was performed and the external jugular vein was isolated and cannulated. A 5F Micropuncture Peel-Away Sheath was advanced into the vein retrogradely to the RV apex over a 4F deflectable catheter under fluoroscopic guidance. A 4F bipolar lead (Medtronic 3830 Secure Select, active fixation, exposed screw) was then advanced through the sheath to the RV apex and screwed in place into the interventricular septum. The lead was connected to a sterilized pulse generator, and both were implanted subcutaneously.

**Fig 1:**
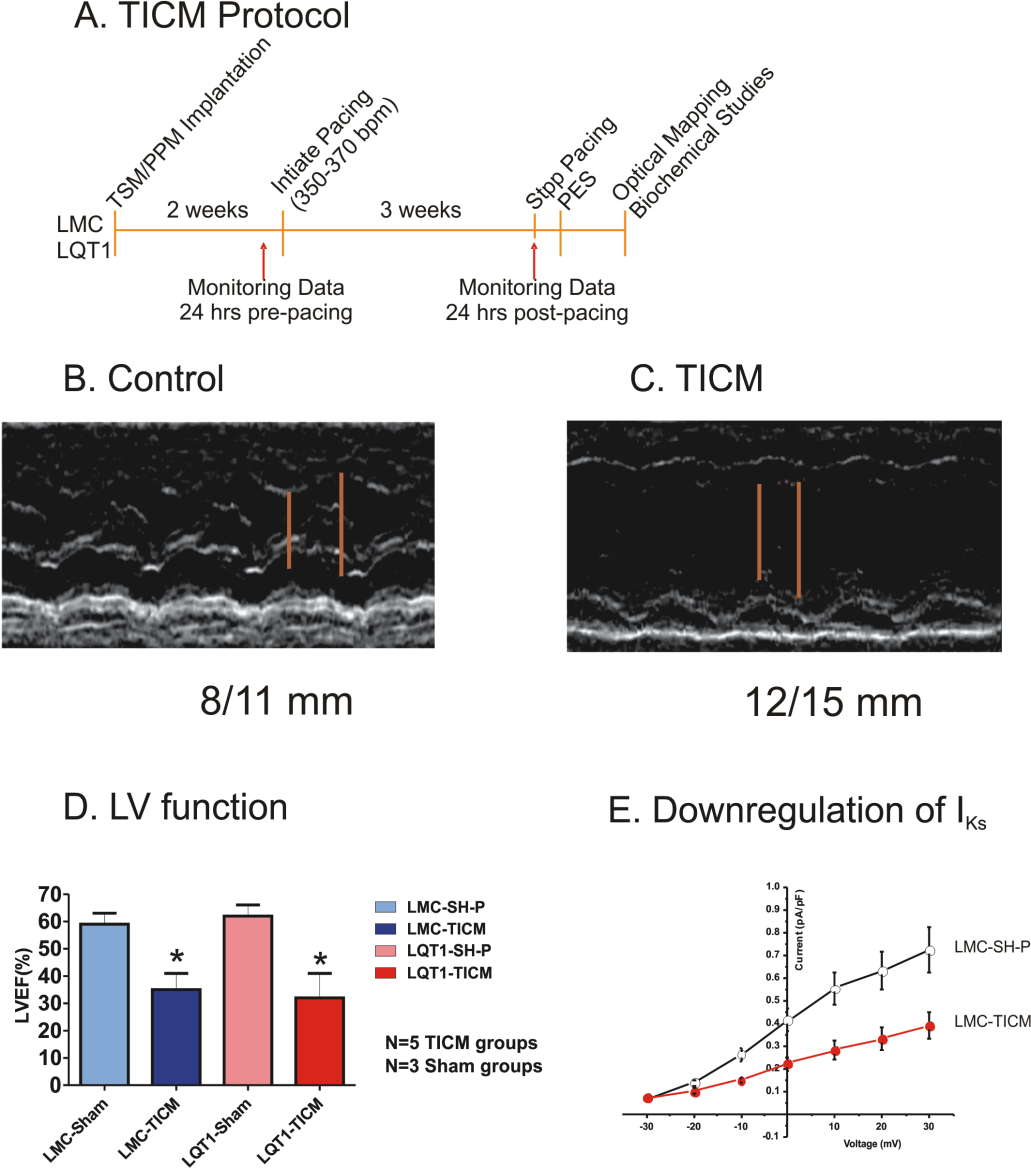
TICM Protocol. (**A**) Three-week pacing protocol. TSM/PPM = transmitter/ pacemaker, PES = programmed electrical stimulation. **(B**) TICM groups show dilated left ventricle and reduced ejection fraction. The red bars indicate end-systolic and end-diastolic LV internal dimensions (8 and 11 mm for LMC sham pacing and 12 and 15 mm for TICM). **(C)** Post-pacing protocol LV function presented as left ventricular ejection fraction in LMC-TICM, LQT1-TICM, and their sham controls. TICM rabbits show statistically significant differences in LV ejection fraction compared with sham; *P<0.05. **(D)** Quantification of I_Ks_ in LMC rabbits. Current amplitude was normalized to cell capacitance. Compared with LMC-SH-P (n = 12), significant downregulation of I_Ks_ was seen in LMC-TICM (n = 11).

Following a week recovery period, the rabbits underwent a three-week rapid pacing protocol (350 bpm for 1 week, then 370 bpm for 2 weeks; see Fig 1A). The two-step stimulation protocol was necessary due to the long refractoriness characteristic of LQT1 rabbits. Following the pacing period, the pacemaker was reprogrammed to OVO nonpacing mode (baseline HR = 240 bpm). The pacemaker implementation was carried out in 34 rabbits; 11 LMC and LQT1 rabbits were paced to induce HF (LMC-TICM and LQT1-TICM), and the remaining 6 LMC and LQT1 rabbits were not paced (sham pacing; SH-P).

### Echocardiography

To assess LV function following the three-week pacing protocol, we performed transthoracic echocardiography in sedated animals (LMC-TICM, LQT1-TICM, n = 5; LMC-SH-P, LQT1-SH-P, n = 3). After anesthesia with ketamine and xylazine, the chests of rabbits were shaved and ECG leads were attached for simultaneous recording of ECG and echocardiography. Two-D echocardiogram images (Hewlett Packard 5500) were obtained using a 7.5 mHz probe, and both long- and short-axis views were used, similar to human echocardiograms. The M-mode was obtained from the short-axis view. Analysis of LV and RV dimensions, left atrium, wall thickness, valve function, and LV ejection fraction (by Simpson’s planimetry method) were performed by a blinded echocardiographer. Fig 1B shows typical examples of 2D echocardiogram images from sham and TICM rabbits. The TICM protocol induced dilated cardiac myopathy after three weeks of pacing.

### Telemetric ECG monitoring, QT/RR ratio

LQT1 and LMC rabbits were monitored using telemetric ECG devices to calculate QT/RR ratios (LMC-TICM, LMC-SH-P, LQT1-SH-P, LQT1-TICM, n = 5) [42]. Telemetric ECG signals were acquired by Dataquest A.R.T. data-acquisition software and analyzed with Ponemah ECG analysis software (both Data Sciences International). QT and RR intervals were measured and averaged over 5 seconds every 20 minutes over 48-hour monitoring periods prior to and following the pacing protocol. Linear regressions of the QT/RR relationships were then performed for each animal both before and after the pacing protocol, and the resulting regressions were then averaged per experimental cohort.

### Minimally invasive in vivo electrophysiological studies (EPS)

The EPS protocol was modified from a previously established protocol from our lab [44]. Studies were performed in the animal electrophysiology (EP) laboratory with a two-channel computer-based programmable stimulator (EP Med systems) and an EP digital recording system (Prucka). LMC-TICM, LQT1-TICM, and their respective sham rabbits were anesthetized, intubated, and monitored as described for pacemaker implantation. A decapolar transvenous 4F electrophysiology catheter (Inquiry, Boston Scientific, Natick, MA) was inserted into the right femoral vein through a 4F sheath and advanced toward the right ventricle, with guidance by fluoroscopy and pacing thresholds. During the procedure, 12-lead surface and five intra-cardiac ECG signals were recorded continuously using EP-Bard-System software OS2/warp (kindly provided by Bard, Lowell, MA, USA). EPS were performed at basic cycle length (CL) of 240 ms. Ventricular effective refractory periods in RV apex and RV septal base position (VERPapex, VERPbase) were analyzed by progressively shortening the S2-interval in 10-ms steps after 10-beat S1 trains. Programmed ventricular stimulation was performed with one, two, and three extra stimuli in apical and basal positions to investigate inducibility of sustained VF. VF frequency was determined by the inverse of the averaged cycle lengths measured during the last five seconds of each VF episode.

### Patch Clamping

Isolation of cardiomyocytes by standard enzymatic techniques and patch-clamp recordings were performed as described previously [42]. Apical ventricular myocytes were isolated from hearts (n = 3 each from LMC-SH-P, LMC-TICM, LQT1-SH-P, and LQT1-TICM groups). Whole-cell recordings (of 11–18 cardiomyocytes per group) were obtained with an Axopatch-200B amplifier (Axon Instruments) with standard patch-clamp techniques. The methods for K^+^ current recording were the same as before [42]. I_Ca,L_ was obtained in Tyrode solution before K^+^ current recording; holding potential was—50 mV, and test potentials were—40 to +40 mV with 10-mV steps lasting 250 ms. I_Ca,L_ was defined as the difference of peak and steady-state current at the end of the pulse. E-4031 (5 μM) and chromanol 293B (30 μM) were used for isolating I_Kr_ and I_Ks,_ respectively. Tetrodotoxin (20 μM) and CdCl_2_ (0.2 mM) were added as needed to block Na^+^ and Ca^2+^ currents.

### Optical Mapping

Rabbits were injected with buprenorphene (0.03 mg/kg IM), acepromazine (0.5 mg^.^kg^-1^ IM), xylazene (15 mg^.^kg^-1^ IM), ketamine (60 mg^.^kg^-1^ IM), pentothal (35 mg^.^kg^-1^ IV), and heparin (200 U^.^kg^-1^). After an appropriate level of anesthesia was achieved, rabbits were euthanized via beating-heart harvest. The hearts were retrogradely perfused through the aorta with (in mmol^.^L^-1^) 130 NaCl, 24 NaHCO_3_, 1.0 MgCl_2_, 4.0 KCl, 1.2 NaH_2_PO_4_, 5 Dextrose, 25 Mannitol, 1.25 CaCl_2_, at pH 7.4, gassed with 95% O_2_ and 5% CO_2._ Hearts were placed in a chamber to maintain temperature, and 5 μmol^.^L^-1^ blebbistatin was added to the perfusate to reduce movement artifact.

The optical apparatus for simultaneous V_m_ and Ca^2+^ recording has been previously described [45]. Sampling rate was set to 1000 frames^.^s^-1^ with 2x2 cm^2^ field of view. Hearts were stained with the voltage-sensitive dye PGH1 [46] (from Dr. Salama at University of Pittsburgh) and calcium-sensitive dye Rhod-2/AM (Invitrogen, Carlsbad).

Hearts were stimulated using a ramp pacing protocol [42, 47] starting from the basic cycle length (CL) of 350 ms to shorter CL with 10-ms steps until either loss of 1:1 capture or VF induction. Ryanodine (2 μM) was perfused for 30 minutes, and the standard stimulation protocol was repeated.

### Data Analysis

The activation and repolarization time-points at each site were determined from fluorescence (F) signals by calculating (dF/dt)_max_ and (d^2^F/dt^2^)_max_. Data was filtered using a spatial Gaussian filter (3×3 pixel), and first/second derivatives (dF/dt, d^2^F/dt^2^) were calculated using a polynomial filter (3^rd^ order, 13 points). Pixels with low signal-to-noise ratio determined by (dF/dt)_max_ (lower than 3×σ of baseline) and outliers of pixels determined by Grubbs’ test were removed from the analysis (typically less than 1% of total pixels) [48]. APD dispersion was defined as APD_max_-APD_min_ [42].

Alternans analysis of APD and Ca^2+^ duration was performed by comparing odd and even beats as described in [49, 50]. The nodal lines of DA, where alternans phase shifts occurred, were identified by detecting pixels with negligible APD difference between odd and even beats as described in [51]. Briefly, the local temporal periodicity of fluorescence signals was recognized using the following equation,

$$\Delta F\left(\vec{r},t\right)=\frac{1}{\tau }\int_{0}^{\tau }\left|F\left(\vec{r},t+t^{\prime }\right)-F\left(\vec{r},t+\tau +t^{\prime }\right)\right|dt^{\prime }$$ 1

where $F(\overset{⃑}{r,t})$ is fluorescence and τ is the pacing cycle length. The pseudo-color images were reconstructed for visualizing $\Delta F(\overset{⃑}{r},t)$. The larger value (or brighter color) of $\Delta F(\overset{⃑}{r},t)$ denotes the region exhibiting alternans, while $\Delta F\left(\overset{⃑}{r},t\right)=0$(or darker color) means no alternans, which displays as nodal lines [51].

### Computer Simulation Methods

Computer simulations were carried out using a ventricular myocyte model modified from the model developed by Restrepo et al.[52]. The sarcolemmal ionic currents were adopted from the model by Mahajan et al. [53] with the L-type Ca^2+^ channels and Na^+^-Ca^2+^ exchange properly distributed in space. The ryanodine receptors and L-type Ca^2+^ channels were simulated using stochastic algorithms. To model LMC-TICM, we reduced I_Ks_ by 50% (see Fig 1E); reduced the maximum SERCA activity by 33% [54, 55] but increased RyR leakiness by doubling the rate constant from the closed state to the open state [54], and doubled the Na^+^-Ca^2+^ exchange activity [26]. To model LQT1-TICM, we further reduced I_Ks_ to zero. Details of the model and computer simulation methods are presented in S1 File.

### Statistical Analysis

For normally distributed values, we used Student’s *t*-test (paired and unpaired) to compare the means of two groups and the Mann-Whitney test to compare values not normally distributed. Fisher’s exact test was used for categorical variables. All data are presented as means ± standard deviation, and a *p* value < 0.05 was considered significant.

## Results

Experiments were carried out in the following sequence: 1) TICM protocol to create HF, 2) echocardiographic studies to verify TICM phenotype in different genotypes, 3) *in vivo* ECG monitoring to examine restitution kinetics from free-moving animals, 4) *in vivo* EPS for VF inducibility, and 5) optical mapping of isolated hearts to investigate VF mechanisms (Fig 1A).

### Rabbit Model of HF

Four cohorts of rabbits were studied: LMC sham pacing (LMC-SH-P), LMC-TICM, LQT1 sham pacing (LQT1-SH-P), and LQT1-TICM. After three weeks of tachypacing, we verified HF phenotypes in TICM rabbits based on LV ejection fraction. TICM rabbits exhibited a significant reduction in mean LV ejection fraction (LVEF (%), n = 5; LMC-SH-P: 59±7; LMC-TICM: 35±6, LQT1-SH-P; 62±7, LQT1-TICM: 32±9, see Fig 1C). Furthermore, the development of HF in tachypaced rabbits was also indicated by ascites, pleural effusions, pericardial effusions, weight increase, and significant reduction in activity.

### Downregulation of I_Ks_ and VF induction during in vivo EPS

**Fig 2:**
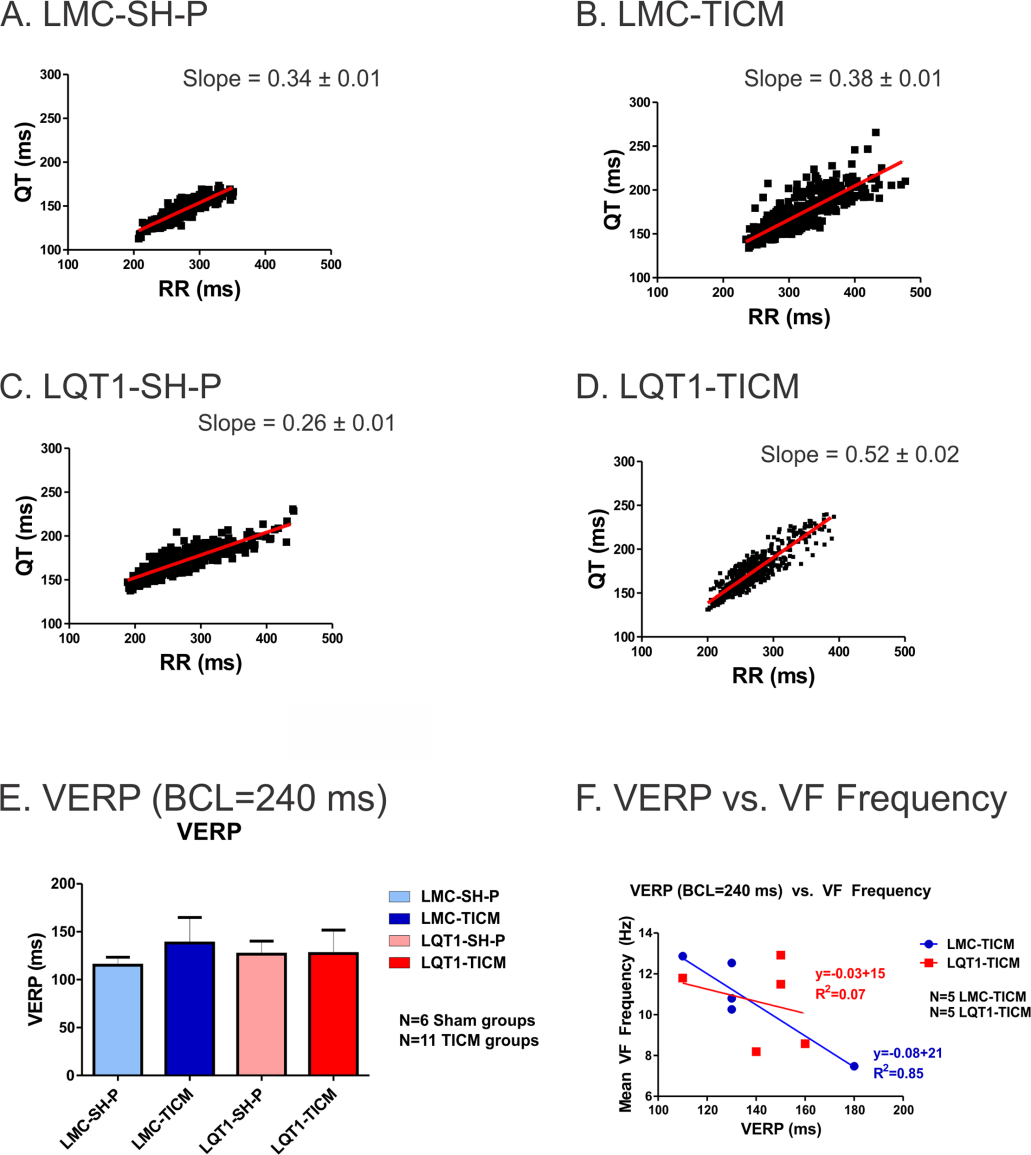
Free-Moving Telemetry: QT-RR Ratios. (**A-D**): QT/RR relationship in awake, free-moving rabbits, recorded approximately every 20 minutes for 24 hours in each group. Lines indicate linear regression derived from the mean of all individual regression lines per genotype. Note that LQT1-TICM demonstrates steeper QT/RR slope post-pacing compared to pre-pacing. (**E**): VERP at BCL = 240 ms in LMC-TICM, LQT1-TICM, and sham animals. No significant difference was found among the four groups (**F**): VERP (BCL = 240 ms) vs. VF frequency in 5 LMC-TICM and LQT1-TICM animals. Unlike LMC-TICM, LQT1-TICM did not show a correlation between VERP and VF frequency.

Cellular electrophysiological study demonstrated that I_Ks_ was significantly downregulated in LMC-TICM (Fig 1**C**) in line with previous studies that showed ~55% downregulation of I_Ks_ [43]. To assess the impact of TICM on cardiac repolarization, we recorded ECGs from free-moving animals and compared the QT/RR ratios before and after the TICM pacing protocol. Free-moving telemetry demonstrated significant steepening of the QT/RR slope post-tachypacing, which was most notable in LQT1-TICM rabbits (pre: 0.26 ± 0.04, post: 0.52 ± 0.02, P<0.05) (Fig 2). Although higher QT/RT slope trends were observed in LMC-TICM (0.34 ± 0.01 vs. 0.38 ± 0.01), the results were not statistically significant.

We investigated refractoriness and vulnerability to VF under *in vivo* EPS using programmed stimulation. VERP in vivo did not reveal significant differences across cohorts (panel E) regardless of progress in deterioration of LV function and the reduction of I_Ks_ in LMC-TICM. Due to the I_ks_-blocking properties of inhaled isoflurane (the anesthetic used for sedation in EPS), VERP measurements here should be interpreted with caution. Both LMC-TICM (3 of 11) and LQT1-TICM (6 of 11) animals were found to be inducible for VF during the programmed stimulation protocol, while sham-operated animals were not inducible (Table 1).

**Table 1:** *In vivo* VF Inducibility using Pen protocol (S1S2S3S4).

Group	+	-
LQT1-TICM	6	5
n = 11		
LQT1-SH-P	0	6
n = 6		
LMC-TICM	3	8
n = 11		
LMC-SH-P	0	6
n = 6		

P<0.05 LQT1-TICM vs. LQT1-SH-P; all other comparisons, P = NS.

VF cycle length has been shown to provide mechanistic insight into VF behaviors [56]. Thus, we measured VF cycle length and correlated baseline VERP measurements to VF. We observed a linear relationship in LMC-TICM, but surprisingly, no correlation between VERP and VF frequency in LQT1-TICM (r = 0.92 vs. r = 0.28, p<0.05, Fig 2F).

### APD dispersion increases in LQT1-TICM

**Fig 3:**
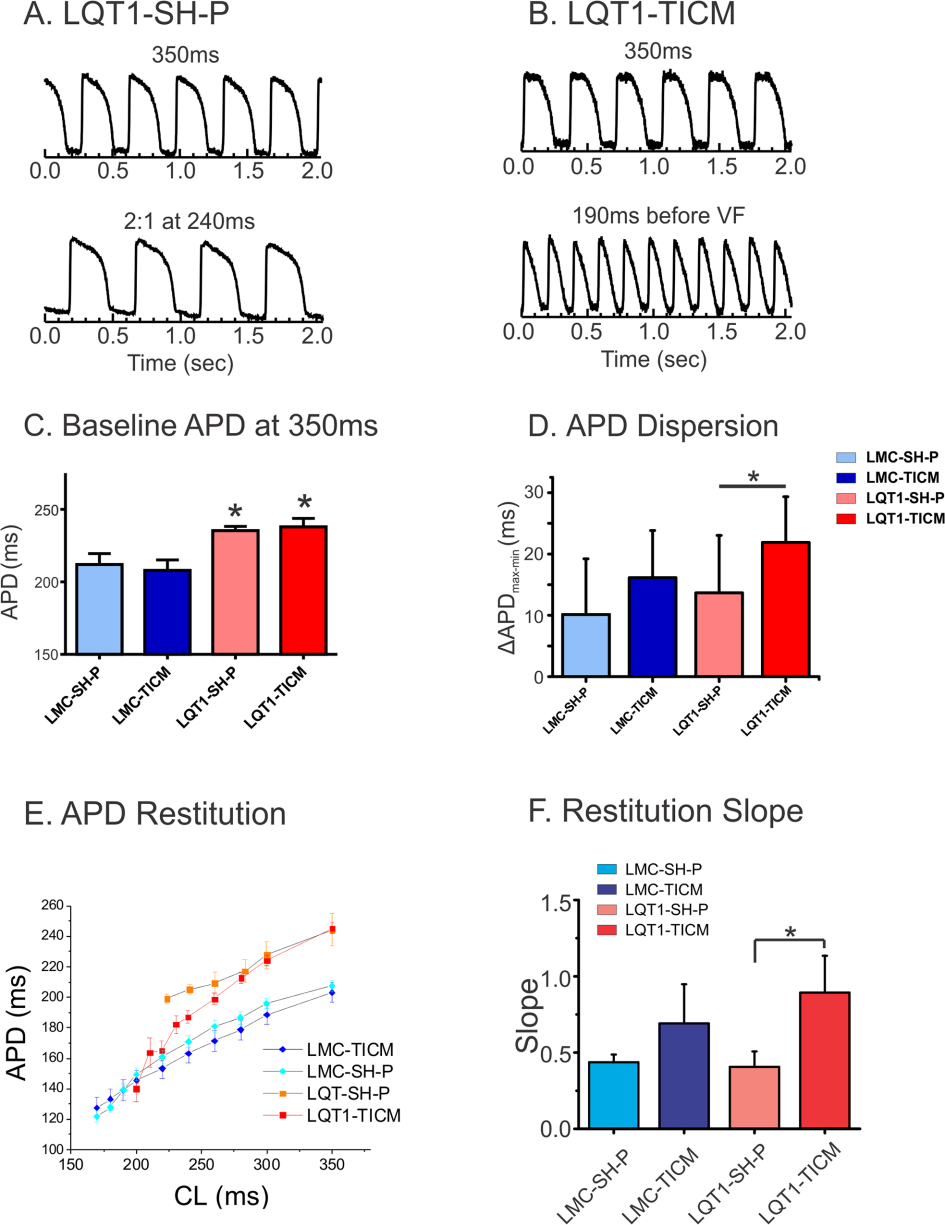
APD dispersion and restitution in TICM rabbits. (**A&B**): Typical raw data of action potential traces from optical mapping. LQT1-SH-P shows 2:1 block at 240 ms CL, while LQT1-TICM was paced as low as 190 ms CL with marked shortening of APD. (**C**) Mean APD in each group at basic cycle length of 350 ms. LQT1 rabbits show statistically significant differences in APD compared with LMC; *P<0.05. (**D**) APD dispersion increased in LQT1-TICM. (**E**) APD restitution curves from four groups. (**F**): Maximum slopes of the APD restitution curves. LQT1-TICM demonstrates increase in APD restitution slopes (*P<0.05).

To further understand the mechanisms underlying the increased arrhythmogenesis in the TICM group, we performed optical mapping experiments. Baseline APD measurements at 350 ms CL revealed significant APD prolongation in LQT1 cohorts as expected (APD in ms, LMC-SH-P: 212±13, LQT1-SH-P: 235±5; n = 5; LMC-TICM: 208±16, LQT1-TICM: 238±13, Fig 3C). Although there was a tendency toward increasing APD dispersion in both TICM models, we found that only LQT1-TICM hearts showed a statistically significant increase in APD dispersion (ΔAPD in ms, LMC-SH-P: 18.6±8.9, LQT1-SH-P: 16.6±4.9; LMC-TICM: 22.0±6.0, LQT1-TICM: 38.0±6.3, Fig 3D).

### Steep APD restitution and alternans in TICM groups

Fig 3E shows the APD restitution curves from four hearts, one representing each group. APD restitution slope was markedly increased in LQT1-TICM (panel F, Slopes; LMC-SH-P: 0.43±0.05, LQT1-SH-P: 0.40±0.26; LMC-TICM: 0.69±0.25; LQT1-TICM: 0.89±0.24, n = 5 in sham and n = 9 in TICM groups, *P<0.05). As predicted by the restitution hypothesis [36], alternans was frequently observed in LQT1-TICM (8 of 9 hearts), which had the steepest restitution curve. In addition, alternans were spatially discordant in LQT1-TICM (7 of 8 hearts), i.e., one location had short-long phase while another had long-short phase. However, the LMC-TICM showed relatively rare cases of DA (2 of 10 hearts).

**Fig 4:**
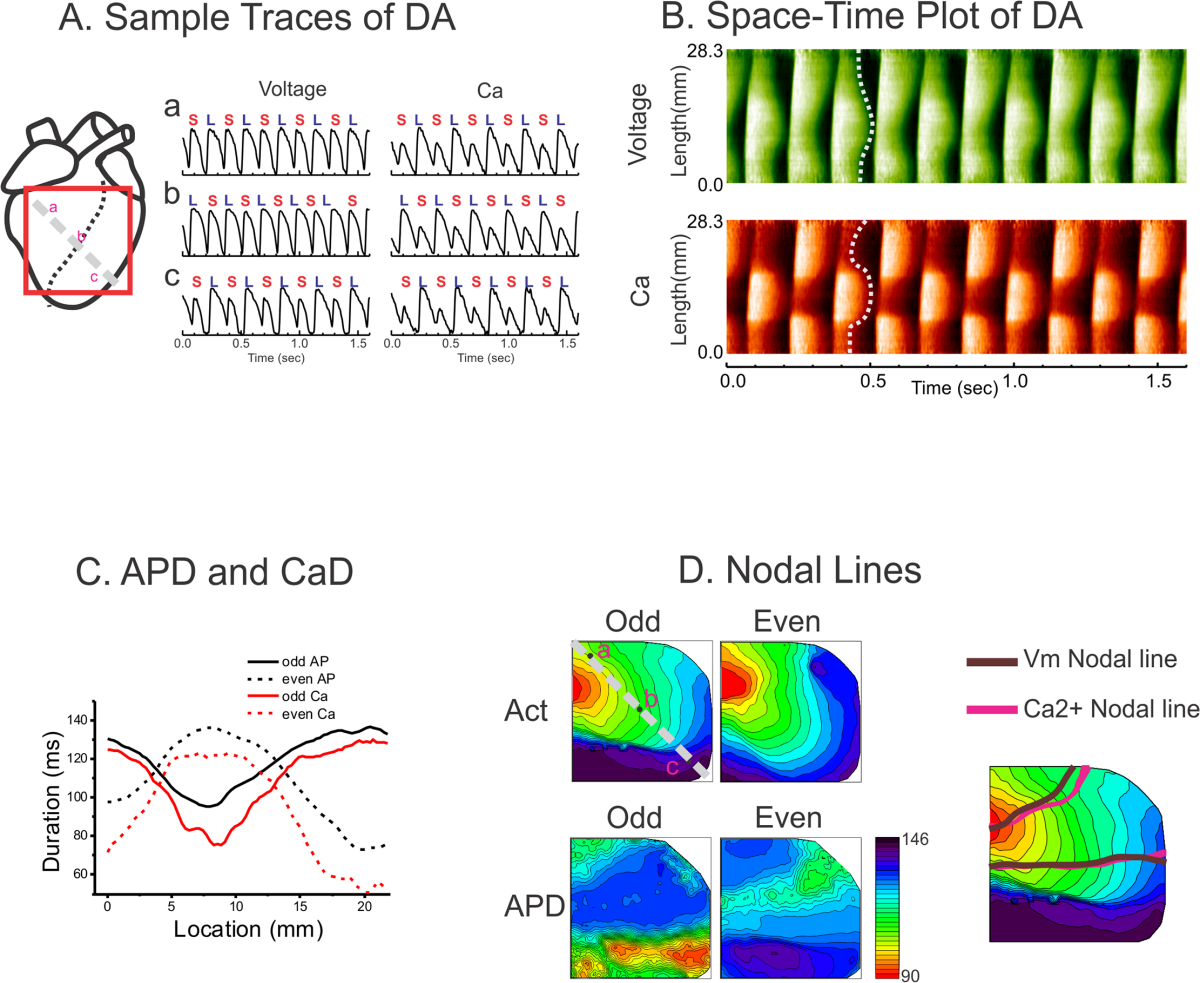
Prevalent discordant alternans in LQT1-TICM. (**A**) Sample traces of DA from three locations. (**B**) Space-time plot of DA along the line a-c in panel A. (**C**) APD (black) and Ca^2+^ duration (red) along the line a-c. Alternation between odd and even beats was larger in Ca^2+^. (**D**) Maps of activation and nodal lines of DA. Note that activation in odd beats shows markedly slowing conduction toward the apex region. However, the nodal lines were not associated with the alternating activation pattern.

Fig 4 shows a typical example of DA in LQT1-TICM. Space-time plots of V_m_ and Ca^2+^ in panel B show that V_m_ repolarization (see dotted white line in panel B) changed gradually in space, while Ca^2+^ transient changed more abruptly. The alternation of Ca^2+^ transient recovery between odd and even beats was greater than that of APD (see panel C). This effect can also be seen in panel A, middle trace (b), where APD alteration between odd and even beats are not prominent, while Ca^2+^ exhibits marked alternation between odd and even beats.

DA can be created by two competing mechanisms: conduction alternans or tissue heterogeneities such as heterogeneous Ca^2+^ handling [57]. Theoretical studies [57] have proposed that the cause of DA can be determined by investigating the behavior of nodal lines (between regions with APD alternans out of phase from each other). The activation maps in Fig 4D indicate that conduction is alternating between odd and even beats. However, the nodal lines superimposed over the activation maps (panel D) are perpendicular to the activation isochronal lines and precede the conduction delay in the odd beats. The conduction delay most likely occurred when the activation front encountered enhanced APD dispersion near nodal lines. This finding suggests that tissue heterogeneities may play an important role in the formation of DA.

### Characteristics of VF in LQT1-TICM

**Fig 5:**
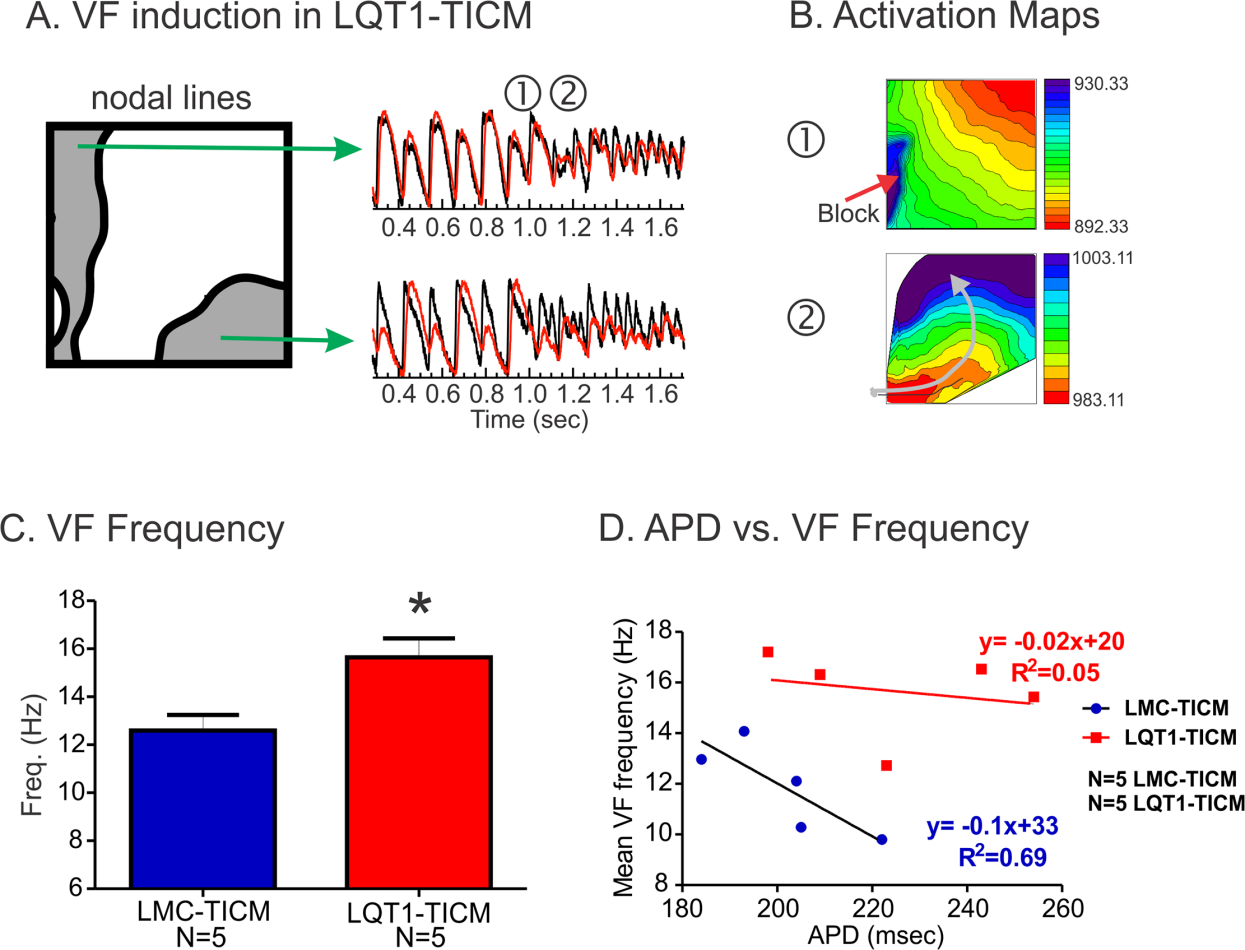
VF induction and VF frequency in LQT1-TICM. (**A**) Sample traces of V_m_ and Ca^2+^ from 190 ms during VF induction. DA was prominent before VF. (**B**) Activation maps of VF induction. Conduction block occurred near the nodal line and formed a rotating wave (grey arrow). (**C**) VF frequency. VF frequency was higher in LQT1-TICM despite the prolongation of APD at basic CL (Fig 3C (**D**) Correlation between APD and VF frequency. The baseline APD is no longer a predictor of VF frequency as in *in vivo* EPS in Fig 2F.

As in *in vivo* experiments, LMC-TICM and LQT1-TICM both demonstrated increased propensity to VF under ramp pacing protocol during optical mapping (6 of 9 LMC-TICM and 7 of 9 LQT1-TICM were inducible; see Table 2). Interestingly, DA often preceded VF induction in LQT1-TICM hearts. Fig 5 shows an example of DA that preceded VF induction. The initiation of VF was due to a conduction block on the left side of the nodal lines (➀) that formed a rotating wave (➁, arrow). This result demonstrates direct link between DA and VF induction in LQT1-TICM.

**Table 2:** VF inducibility in optical mapping.

Group	VF	No VF
LQT1-TICM	7	2
n = 9		
LQT1-SH-P	1	4
n = 5		
LMC-TICM	6	3
n = 9		
LMC-SH-P	2	3
n = 5		

LQT1-TICM vs. LQT1-SH-P: p = 0.09

VF frequency in LQT1-TICM was significantly higher in LQT1-TICM than in LMC-TICM (Frequency (Hz), n = 5; LMC-TICM: 12.59±1.47, LQT1-TICM: 15.65±1.75, *P<0.05, panel C). The basic relationship between baseline APD and VF frequency was lost (r = 0.92 vs. r = 0.28, p<0.05, panel D) as in *in vivo* EPS (Fig 2F).

### Lack of I_Ks_ leads to greater APD alternans in computer simulation of HF

**Fig 6:**
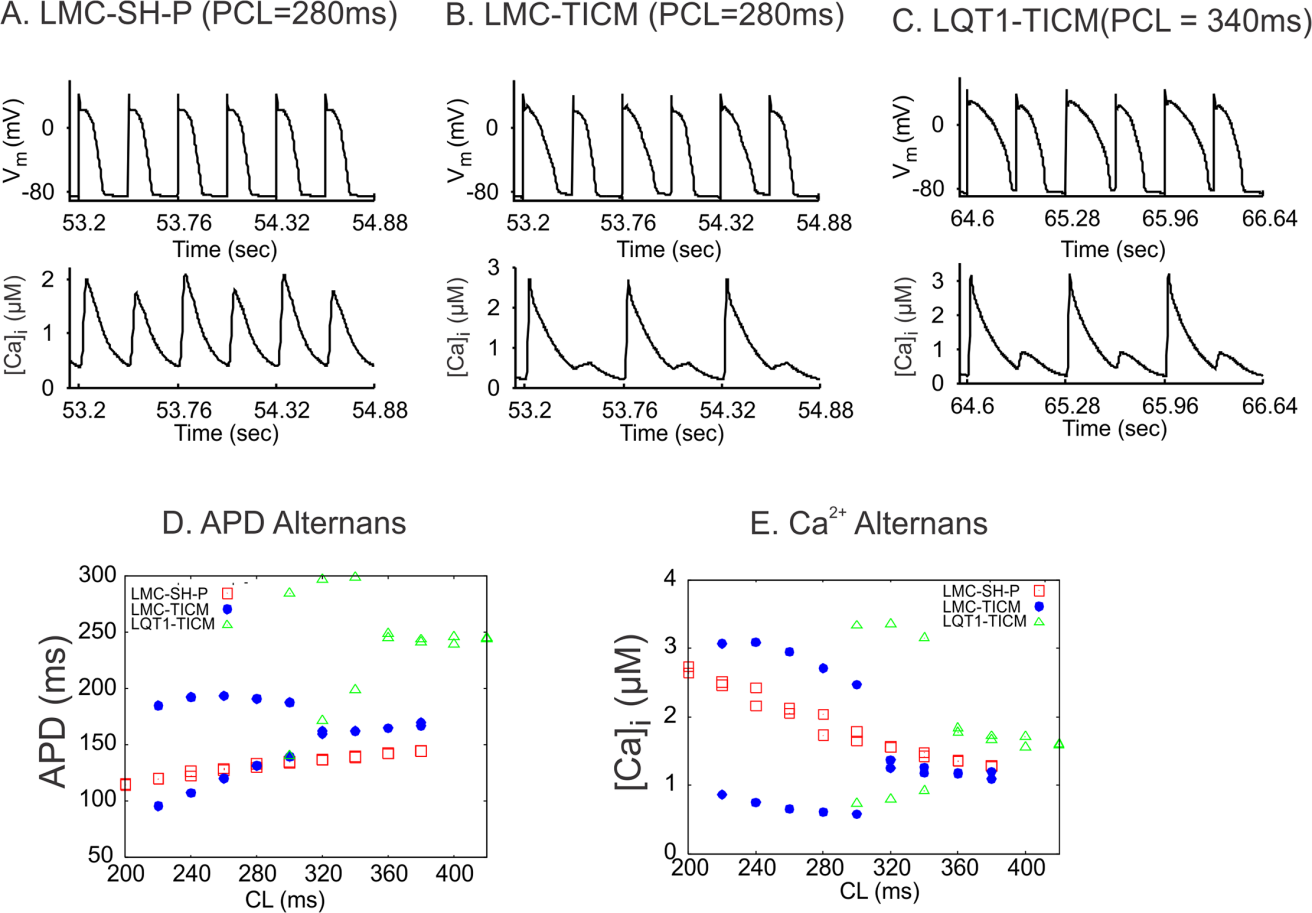
Computer simulations of APD and Ca2+ alternans under different conditions. **(A**) V_m_ (upper) and whole-cell Ca^2+^ concentration (lower) versus time for the control condition at CL = 280 ms. (**B**) V_m_ and whole-cell Ca^2+^ concentration versus time for the HF condition. (**C**) V_m_ and whole-cell Ca^2+^ concentration versus time for the same condition as in B but with zero I_Ks_. (**D**) Peak whole-cell Ca^2+^ concentrations of two consecutive beats versus CL for the three conditions. (**E**) APD of the same two consecutive beats as in D versus CL for the three conditions.

**Fig 6** shows the results of a computer model of ventricular myocytes, which includes a detailed spatiotemporal Ca^2+^ cycling system (details are provided in S1 File). Under the control condition, both APD and Ca^2+^ exhibited very small amplitudes of alternans at rapid pacing rates. However, under HF conditions, large-amplitude alternans of APD and Ca^2+^ occurred when the pacing cycle length was shorter than 320 ms. Alternans in the HF condition was caused mainly by changes in Ca^2+^ cycling properties from the control condition, i.e., Ca^2+^ cycling was the major origin of alternans [58]. With further reduction of I_Ks_ from the HF condition to mimic the LQT1-TICM condition, alternans occurred at pacing cycle length shorter than 360 ms, and the amplitudes of APD and Ca^2+^ alternans were further increased. In other words, alternans was further potentiated by the lack of I_Ks_ in addition to remodeling of Ca^2+^ cycling in HF. This supports the experimental observation that LQT1-TICM rabbits had a higher propensity to alternans and arrhythmias than LMC-TICM rabbits.

### Effects of ryanodine on the behavior of DA in LQT1-TICM

**Fig 7:**
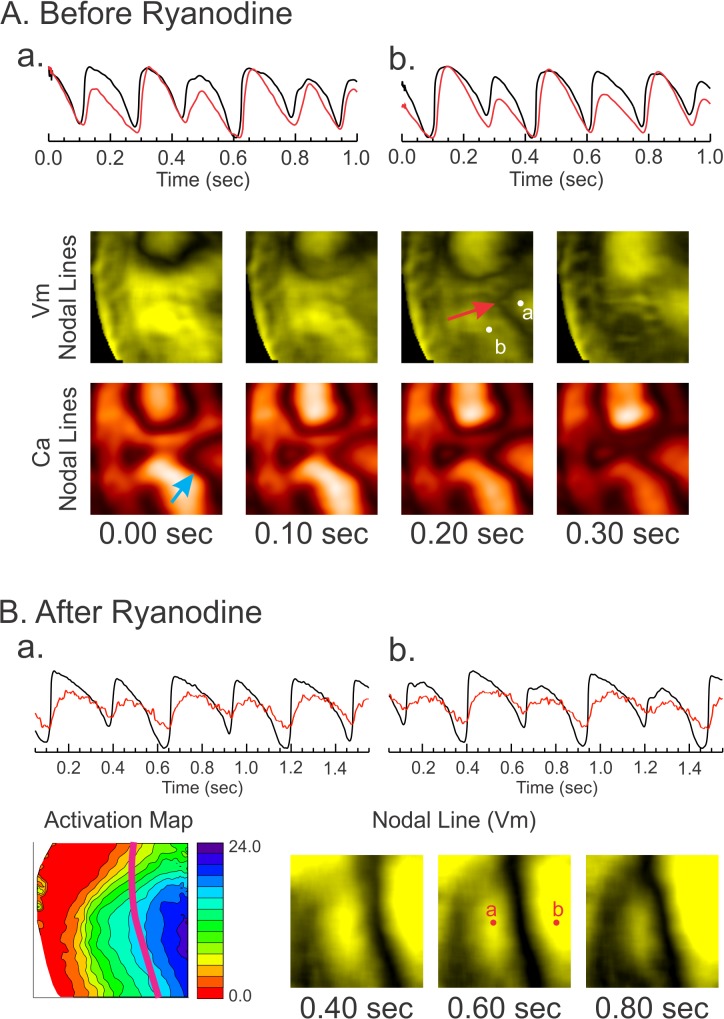
The effect of ryanodine on DA. (**A**) Complex Ca^2+^ DA in LQT1-TICM. *Top*: V_m_ and Ca^2+^ traces from three locations. Note that the phase shift from short-long to long-short occurs in Ca^2+^, which was followed by V_m_. *Bottom*: Series of nodal line images. Initially, a single nodal line was observed in V_m_, while an additional nodal line was seen in Ca^2+^ (blue arrow). Several beats later, a nodal line appeared in V_m,_ which is in close proximity to the Ca^2+^ nodal line (red arrow). (**B**) Nodal line behavior after 2 μM ryanodine. DA were observed in only one third of LQT1-TICM hearts with ryanodine (3 of 9 hearts). The series of nodal line images shows that the beat-to-beat changes in DA were minimal and closely related to the activation pattern.

In an effort to investigate the potential role of Ca^2+^ underlying the increased propensity to DA in LQT1-TICM, Ca^2+^ transients were abolished by 2 μM ryanodine, and the ramp pacing protocol was repeated. Before ryanodine administration, the behavior of DA was complex, dynamically appearing and disappearing (see Fig 7). Importantly, Ca^2+^ nodal lines were independent of APD nodal lines, often appearing without them. In panel A, additional Ca^2+^ nodal lines appear alone (1^st^ column, blue arrow), followed by formation of the APD nodal line (3^rd^ column), suggesting that Ca^2+^ instability promotes complex V_m_ DA. As expected, abolishing Ca^2+^ transient with ryanodine reduced the incidence of DA (6 of 9) and VF induction in LQT1-TICM. Some LQT1-TICM hearts still demonstrated DA under ryanodine (3 of 9 hearts), though their dynamics were markedly different before ryanodine perfusion. The nodal lines of V_m_ DA under ryanodine were closely related to the activation pattern (Fig 7B) and slowly moved toward the pacing site, which was predicted by computer modeling studies when the conduction velocity restitution causes DA. Overall, our data provide strong evidence that the lack of I_Ks_ combined with abnormal Ca^2+^ handling increase voltage instability and arrhythmia risk in failing hearts.

## Discussion

We evaluated the effects of I_Ks_ remodeling on arrhythmogenesis in HF using a transgenic rabbit model of LQT1 and their littermate controls. Our major findings are that while the HF phenotype is very similar between LMC-TICM and LQT1-TICM, the complete lack of I_Ks_ in LQT1-TICM produces a more arrhythmogenic substrate due to complex APD and Ca^2+^ DA, ultimately causing conduction block and VF.

### Steep restitution and alternans in TICM

We previously reported that rabbit LQT1 hearts have smaller APD dispersion and a flatter restitution curve than LMC [42]. Ramp pacing and programmed stimulation were less effective in inducing alternans and VF in LQT1 rabbits, suggesting that lack of I_Ks_ alone does not promote alternans reentry [42, 50]. In contrast, TICM protocol steepened APD restitution curves in LQT1 both *in vivo QT/RR* plot (Fig 2) and *ex vivo* optical mapping studies (Fig 3). In line with APD restitution theory, LQT1-TICM demonstrated higher risk of DA and VF induction under programmed stimulation (Table 1). VF initiation (Fig 5) indicates that conduction block occurred in the region of the large repolarization gradient caused by DA, verifying alternans as a major factor behind arrhythmogenesis in LQT1-TICM.

It is well known that APD restitution is steep in animal models and human HF [8, 59–61], but our result is quite surprising, because the lack of I_Ks_ in LQT1-TICM is supposed to prevent arrhythmogenic repolarization shortening and protect against reentry formation [37, 38]. Since electrical remodeling in LQT1-TICM is relatively minor (see S2 File for ionic current remodeling; no statistical significance was found), our results suggest that the combination of I_Ks_ downregulation and remodeling in Ca^2+^ handling in the failing heart plays a major role in inducing alternans and reentry formation.

### I_Ks_ downregulation accentuates Ca^2+^-mediated alternans in TICM

Abnormal Ca^2+^ handling in HF has been well documented and is thought to play a key role in arrhythmogenesis [4, 25, 62, 63]. Action-potential clamp studies showed that Ca^2+^ transients can still alternate in isolated myocytes from failing hearts under non-alternating action potential clamp conditions [64], indicating that Ca^2+^ alternans are independent from electrophysiological remodeling of repolarizing currents in HF. Ca^2+^ alternans in our data was much larger than APD alternans (Fig 4A) and also changes its phase abruptly across nodal lines (see Fig 4B & 4C), while APD alternans gradually changes across nodal lines. In addition, Ca^2+^ alternans was still present in certain locations even when APD alternans was not prominent (Fig 4A and 4B and S4 File and S5 File), indicating that abnormal Ca^2+^ handling is a major driver of APD alternans in HF. Ryanodine significantly reduced the incidence of DA and VF in the LQT1-TICM group, demonstrating that Ca^2+^ is a major driver of DA in the failing heart.

Ca^2+^ alternans in HF can promote APD alternans through Ca^2^-depedendent ionic currents. For example, I_NCX_ is upregulated in HF [25–28], which can enhance APD alternans via alternating depolarizing currents during Ca^2+^ alternans. Our experimental and computer simulation data suggest that I_Ks_ downregulation is also an important factor to accentuate APD alternans. Since I_Ks_ is Ca^2+^ dependent and is augmented by Ca^2+^ transients, the reduced I_Ks_ in HF may increase the influence of Ca^2+^ on V_m_ through I_NCX_, resulting in enhanced APD alternans.

### Complex DA dynamics in TICM

In the present study, LQT1-TICM lacking I_Ks_ demonstrated highly dynamic Ca^2+^ DA, changing from its phase of alternans (S3 File and S1 Movie). Detailed analysis shows that the phase transition of Ca^2+^ alternans often precedes that of APD alternans (see S4 File). As a result, more complex APD nodal lines were readily formed in LQT1-TICM hearts. The complex nodal lines increased the risk of conduction block due to increased dispersion of repolarization. In this study, complex nodal line dynamics were observed almost exclusively in LQT1-TICM (see Fig 7 and S3 and S4 Files for LQT1-TICM vs. S5 File for LMC-TICM), suggesting the role of I_Ks_ downregulation along with abnormal Ca^2+^ handling in HF.

It is important to point out that a small number of LQT1-TICM hearts (3 of 9 LQT1-TICM) still exhibited DA after ryanodine perfusion. The nodal lines after ryanodine administration indicate abnormal conduction, as they are aligned with wave fronts and move towards the pacing sites, unlike the case in LQT1-TICM (Fig 7). Abnormal conduction is another well-known phenomenon in HF due to fibrosis, gap junction remodeling, and downregulation of I_Na_ [4, 62, 65]. Our result shows the complex nature of HF and demonstrates that multiple arrhythmia mechanisms, including Ca^2+^ alternans and slow conduction, contribute to overall arrhythmia risk in HF.

### High-frequency VF in LQT1-TICM

Our study demonstrated that VF in LQT1-TICM exhibited significantly higher VF frequency despite prolonged APD. Since LQT1-TICM hearts lack I_Ks_, it is surprising to see even higher-frequency VF in LQT1-TICM compared to LMC-TICM. The higher VF frequency in LQT1-TICM may be related to its steep APD restitution. As a result, refractoriness and wavelength can be substantially shortened at short CLs such as in VF, allowing high-frequency VF in LQT1-TICM. The cause of APD shortening at rapid heart rate is not clear. Harada et al.[8] provided mechanistic insight into APD shortening in the failing heart, linking it to downregulation of I_CaL_. Further studies are needed to understand the mechanism underlying APD shortening and high-frequency VF in LQT1-TICM.

### Conclusions

Our results strongly support the contention that lack of repolarization reserve in HF, particularly reduction in I_Ks_, is highly arrhythmogenic. Abnormal Ca^2+^ handling in failing hearts promotes Ca^2+^ alternans, and lack of I_Ks_ enhances the effect of Ca^2+^ alternans on APD alternans, complicating the dynamics of DA and VF. This study emphasizes that the repolarization reserve provided by I_Ks_ is an important modulator of Ca^2+^-driven DA and VF.

## Supporting Information

S1 File Supporting information for computer modeling. (DOCX)

S2 File Electrical remodeling in TICM. (DOCX)

S3 File Rate-dependent changes in V_m_ and Ca^2+^ discordant alternans in LQT1-TICM. (DOCX)

S4 File V_m_ and Ca^2+^ relationship during the phase transition of alternans. (DOCX)

S5 File Discordant alternans and nodal line dynamics in LMC-TICM. (DOCX)

S1 Movie Complex nodal line dynamics in LQT1 TICM. Top panels: V_m_ and Ca^2+^. Bottom panels: V_m_ and Ca^2+^ nodal lines. The nodal lines of LQT1-TICM demonstrated complex patterns including genesis, vanishing, wiggling, and moving. The spatiotemporal patterns of nodal lines of action potential and Ca^2+^ transient were also complex. This movie corresponds to Fig 7A in the manuscript.
(MP4)

S2 Movie Nodal line dynamics after ryanodine. Left panel: V_m_, Right panel: V_m_ nodal line in LQT1-TICM heart after ryanodine. This movie corresponds to Fig 7B in the manuscript.
(MP4)
