# Correction: Spatially Discordant Alternans and Arrhythmias in Tachypacing-Induced Cardiac Myopathy in Transgenic LQT1 Rabbits: The Importance of I_Ks_ and Ca^2+^ Cycling

**DOI:** 10.1371/journal.pone.0132198

**Published:** 2015-07-01

**Authors:** Emily Lau, Konstantinos Kossidas, Tae Yun Kim, Yukiko Kunitomo, Ohad Ziv, Zhen Song, Chantel Taylor, Lorraine Schofield, Joe Yammine, Gongxin Liu, Xuwen Peng, Zhilin Qu, Gideon Koren, Bum-Rak Choi

The sixth author’s name is spelled incorrectly. The correct name is: Zhen Song. The correct citation is: Lau E, Kossidas K, Kim TY, Kunitomo Y, Ziv O, Song Z, et al. (2015) Spatially Discordant Alternans and Arrhythmias in Tachypacing-Induced Cardiac Myopathy in Transgenic LQT1 Rabbits: The Importance of I_Ks_ and Ca^2+^ Cycling. PLoS ONE 10(5): e0122754. doi:10.1371/journal.pone.0122754
