# Supplementary material for: Spatially Discordant Alternans and Arrhythmias in Tachypacing-Induced Cardiac Myopathy in Transgenic LQT1 Rabbits: The Importance of IKs and Ca2+ Cycling
Source: PLoS One. 2015 May 13;10(5):e0122754. doi: 10.1371/journal.pone.0122754 (PMC4430457; doi:10.1371/journal.pone.0122754)
Supplement: S1 File — (DOCX) [file pone.0122754.s001.docx]

### Computer model and parameter set

Computer simulations were performed using a rabbit ventricular moycyte model that was constructed by combining mathematical formulations of selected sarcolemmal currents from Mahajan et al. ^1^, together with a mathematical model of Ca handling from Restrepo et al. ^2^. Fig S1 shows the schematics of the model (Aa and Ab) and the action pontential, calcium transient, and a line scan of the calcium transient (B).


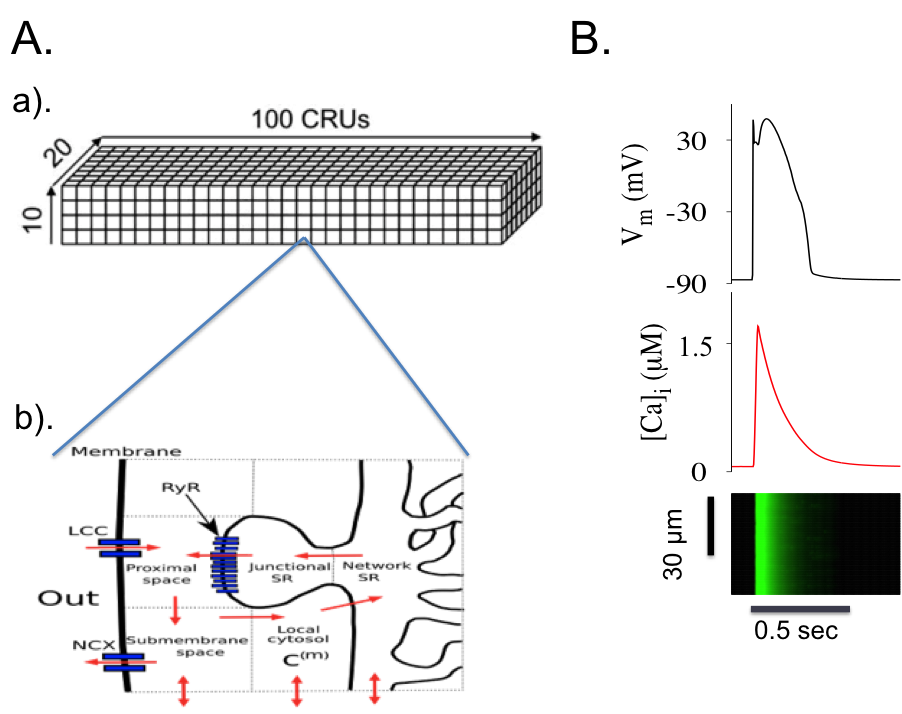


**S1 Fig.** A. a). Myocytes are simulated by a network of 20,000 diffusively coupled calcium release units (CRUs) arranged on a 3-d grid. A. b). Each unit consists of local cytosolic, submembrane, proximal, JSR, and NSR compartments. L-type Ca channels release Ca^2+^ into the proximal space and the sodium-calcium exchange current acts on the submembrane space. The red arrows illustrate the average direction of Ca^2+^ flow. B). Time traces of membrane potential and Ca^2+^ transient, and the corresponding line scan image, where the line was taken along the center axis in the longitudinal direction.

The dynamics of membrane voltage, V_m_, is described by the equation

$\frac{dV_{m}}{dt}= - \frac{1}{C_{m}}(I_{ion}+I_{stim}$) (1)

where $I_{\mathrm{ion}}$ is the total membrane current density, $I_{\mathrm{stim}}$is the stimulus current, and where $C_{m}$ is the cell membrane capacitance. Following Mahajan et al. ^1^, all ion currents are computed for 1μF of cell membrane capacitance and have units of μA/μF.

The total membrane current is given by

$I_{ion}= I_{Na}+ I_{to}+I_{Kr}+I_{Ks}+I_{K1}+I_{NaK}+I_{Ca}+I_{NaCa}$ (2)

Ca cycling was simulated using a model developed by Restrepo et al ^2^. The equations for Ca cycling are:

$\frac{dC_{i}}{dt}=\beta_{i}\left( c_{i} \right)\left( J_{dsi}\frac{v_{s}}{v_{i}}+ J_{up}+J_{leak}-J_{TCi}+J_{ci} \right),$ (3)

$\frac{dC_{s}}{dt}=\beta_{s}\left( c_{s} \right)\left( {J_{dps}\frac{v_{p}}{v_{s}}+J}_{NCX}-J_{dsi}-J_{TCs}+J_{cs} \right),$ (4)

$\frac{dC_{p}}{dt}=\beta_{p}\left( c_{p} \right)\left( {J_{r}+J}_{Ca}-J_{dps} \right),$ (5)

$\frac{dC_{nsr}}{dt}=\left( {{(J}_{up}-J_{leak})\frac{v_{i}}{v_{nsr}}-J}_{tr}\frac{v_{jsr}}{v_{nsr}}+J_{cnsr} \right),$ (6)

$\frac{dC_{jsr}}{dt}=\beta_{jsr}\left( c_{jsr} \right)\left( J_{tr}-J_{r}\frac{v_{p}}{v_{jsr}} \right).$ (7)

where$c_{s}$, $c_{i}$, $c_{p}, c_{\mathrm{jsr}}$, and $c_{\mathrm{nsr}}$ are the average concentration of free Ca in submembrane space, the cytosol, the proximal space, the junctional SR, and the network SR, with volumes $v_{s}$, $v_{i}, v_{p}, v_{\mathrm{jsr}},$and $v_{\mathrm{nsr}},$respectively. The factors $\beta_{i}$ and $\beta_{s}, and \beta_{p}$describe instantaneous buffering to Calmodulin, SR sites, Myosin (Ca), and Myosin (Mg). The factor $\beta_{\mathrm{jsr}}$ describes luminal buffers, and the details can be refered in Restrepo et al.

All Ca fluxes have units of $\mu M/ms$, which can be converted to units of $\mu A/\mu F$ using the conversion factor $Fv_{i}/C_{m}$, where F is Faraday’s constant. Thus, ionic fluxes can be converted to currents using

$I_{Ca}=-2\alpha J_{Ca}$ , $I_{NaCa}=\alpha J_{NaCa}$, (8)

where the ion currents are in units of $\mu A/\mu F$. The detailed formulation for the current fluxes can be found in Mahajan et al. ^1^

Modifications of model parameters

We modified the conductance of NCX^3-6^ and parameters in SERCA formula based on previous publications^7, 8^ as shown in Table 1.

**S1 Table. Modifications of parameters from the original model for control condition.**

|  | Original parameters | Modified parameters |
| --- | --- | --- |
| NCX conductance *ν_NaCa_* | 21 μM/ms | 7 μM/ms |
| SERCA conductance ν_up_ | 0.36 μM/ms | 1.08 μM/ms |
| SERCA sensitivity *K_i_* | 0.123 μM | 0.3 μM |

To mimic heart failure condition, we reduced $g_{\mathrm{Ks}}$ by 50%, and further reduced to 0 to mimic heart failure in LQT condition as shown in Table 2. The transition rates from the closed to the open states are assumed to depend on the proximal Ca concentration for the calsequestrin (CSQN)-unbound and CSQN-bound states, respectively, as

$k_{12}=K_{u}c_{p}^{2},$ (9)

$k_{43}=K_{b}c_{p}^{2}.$ (10)

We doubled the rate constants of K_u_ and K_b_ to mimic the effect of heart failure condition.

**S2 Table. Modifications of parameters under different simulation conditions.**

|  | Ctrl | HF | LQT&HF |
| --- | --- | --- | --- |
| SERCA conductance *ν_up_* | 1.08 μM/ms | 0.72 μM/ms | 0.72 μM/ms |
| SERCA sensitivity *K_i_* | 0.3 μM | 0.123 μM | 0.123 μM |
| RyR rate *K_u_* | 0.00038 μM^-2^ms^-1^ | 0.00076 μM^-2^ms^-1^ | 0.00076 μM^-2^ms^-1^ |
| RyR rate *K_b_* | 0.00005 μM^-2^ms^-1^ | 0.0001 μM^-2^ms^-1^ | 0.0001 μM^-2^ms^-1^ |
| NCX conductance *ν_NaCa_* | 7 μM/ms | 14 μM/ms | 14 μM/ms |
| I_Ks_ conductance *g_Ks_* | 0.1386 mS/mF | 0.0693 mS/mF | 0 mS/mF |

## References:

1. Mahajan A, Shiferaw Y, Sato D, Baher A, Olcese R, Xie L-H, Yang M-J, Chen P-S, Restrepo JG, Karma A, Garfinkel A, Qu Z, Weiss JN. A rabbit ventricular action potential model replicating cardiac dynamics at rapid heart rates. *Biophys. J.* 2008;94:392-410

2. Restrepo JG, Weiss JN, Karma A. Calsequestrin-mediated mechanism for cellular calcium transient alternans. *Biophys. J.* 2008;95:3767-3789

3. Pogwizd SM, Schlotthauer K, Li L, Yuan W, Bers DM. Arrhythmogenesis and contractile dysfunction in heart failure: Roles of sodium-calcium exchange, inward rectifier potassium current, and residual beta-adrenergic responsiveness. *Circ Res*. 2001;88:1159-1167

4. Bers DM, Pogwizd SM, Schlotthauer K. Upregulated Na/Ca exchange is involved in both contractile dysfunction and arrhythmogenesis in heart failure. *Basic Res Cardiol*. 2002;97 Suppl 1:I36-42

5. Reinecke H, Studer R, Vetter R, Holtz J, Drexler H. Cardiac Na+/Ca2+ exchange activity in patients with end-stage heart failure. *Cardiovasc Res*. 1996;31:48-54

6. Studer R, Reinecke H, Bilger J, Eschenhagen T, Bohm M, Hasenfuss G, Just H, Holtz J, Drexler H. Gene expression of the cardiac Na(+)-Ca2+ exchanger in end-stage human heart failure. *Circ Res*. 1994;75:443-453

7. Ai X, Curran JW, Shannon TR, Bers DM, Pogwizd SM. Ca2+/calmodulin-dependent protein kinase modulates cardiac ryanodine receptor phosphorylation and sarcoplasmic reticulum Ca2+ leak in heart failure. *Circ Res*. 2005;97:1314-1322

8. Shannon TR, Wang F, Bers DM. Regulation of cardiac sarcoplasmic reticulum Ca release by luminal [Ca] and altered gating assessed with a mathematical model. *Biophys J*. 2005;89:4096-4110
