# Supplementary material for: Spatially Discordant Alternans and Arrhythmias in Tachypacing-Induced Cardiac Myopathy in Transgenic LQT1 Rabbits: The Importance of IKs and Ca2+ Cycling
Source: PLoS One. 2015 May 13;10(5):e0122754. doi: 10.1371/journal.pone.0122754 (PMC4430457; doi:10.1371/journal.pone.0122754)
Supplement: S2 File — (DOCX) [file pone.0122754.s002.docx]

### Electrophysiological remodeling in TICM rabbits

Patch clamping experiments were performed to study ion channel remodeling in LMC-TICM and LQT1-TICM for potential mechanisms of enhanced alternans in LQT1-TICM model. Electrical remodeling was pronounced in LMC group, which includes downregulation of including I_kr_, I_Ks,_ and I_Ca_ (see Figure S4). In contrast, LQT1-TICM did not show significant changes compared to LQT1-sham group. It is interesting to point out that LQT1-sham group already shows downregulation of I_Kr_­ and I_Ca_ compared to LMC-sham group and tachypacing did not further downregulate I_Kr_ and I_­Ca_ in LQT1.


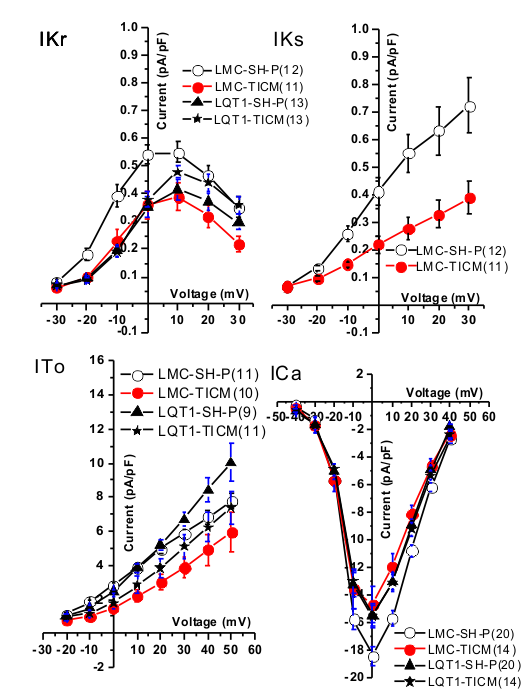


**S2 Fig.** **Mean current density vs. voltage relationships from LMC-SH-P, LMC-TICM, LQT1-SH-P, and LQT1-TICM groups.** The error bars indicate SEM and the number of cells measured are in the parenthesis.
