# Supplementary material for: Spatially Discordant Alternans and Arrhythmias in Tachypacing-Induced Cardiac Myopathy in Transgenic LQT1 Rabbits: The Importance of IKs and Ca2+ Cycling
Source: PLoS One. 2015 May 13;10(5):e0122754. doi: 10.1371/journal.pone.0122754 (PMC4430457; doi:10.1371/journal.pone.0122754)
Supplement: S3 File — (DOCX) [file pone.0122754.s003.docx]

### The rate dependent nodal line dynamics in LQT1-TICM

The discordant alternans in LQT1-TICM were highly dynamic and Ca^2+^ discordant alternans often precedes V_m_ alternans. Figure S5 shows a typical example of complex nodal line dynamics. At cycle length of 200 ms, Ca^2+^ discordant alternans appeared without V_m_ alternans. The nodal line of Ca^2+^ was not associated with V_m_ activation pattern. At 190 ms pacing, another nodal line appeared at the base of LV in V_m_ and Ca^2+^ maps. However, the same Ca^2+^ nodal line appeared at 200 ms was still missing in the V_m_ map. Further shortening of pacing cycle length to 160 ms caused highly complex V_m_ and Ca^2+^ nodal lines with certain nodal lines associated with conduction pattern. Eventually, the stimulation caused VF induction and high frequency VF as described in the Result section.


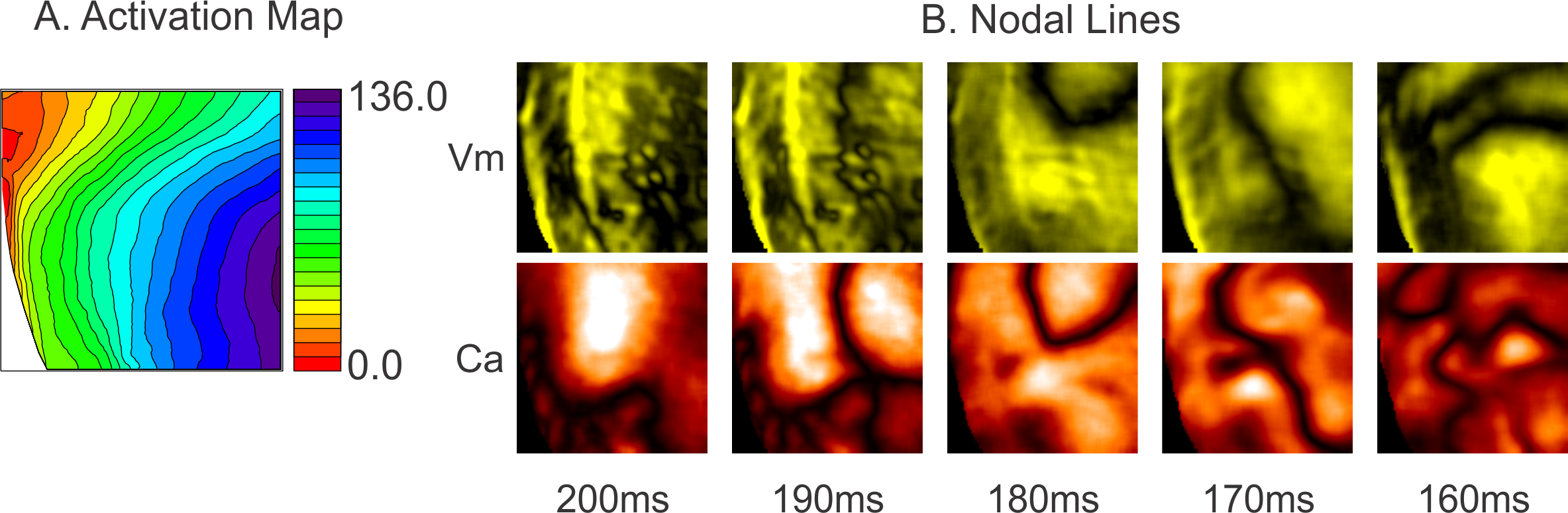


**S3 Fig. Rate-dependent changes in V_m_ and Ca^2+^ discordant alternans in LQT1-TICM.** The heart was stimulation from the RV base and the activation map at 200 ms is shown in the left panel. The pacing cycle length corresponding nodal line maps are noted at the bottom of each image.
