# Supplementary material for: Spatially Discordant Alternans and Arrhythmias in Tachypacing-Induced Cardiac Myopathy in Transgenic LQT1 Rabbits: The Importance of IKs and Ca2+ Cycling
Source: PLoS One. 2015 May 13;10(5):e0122754. doi: 10.1371/journal.pone.0122754 (PMC4430457; doi:10.1371/journal.pone.0122754)
Supplement: S4 File — (DOCX) [file pone.0122754.s004.docx]

### V_m_ and Ca^2^ during the transition of alternan phase from short-long to long-short

An example of the phase transition in LQT1-TICM is shown in Figure S6. It started with beat to beat changing V_m_ and Ca^2+^ and later settled alternans. The settling period is marked with a green box in panel A and corresponding APD and Ca^2+^ duration are plotted in panel B. Right before the onset of the alternans, V_m_ and Ca^2+^ durations vigorously change without clear pattern of alternans followed by small amplitude alternans (green bar). Note that Ca^2+^ alternan phase settled earlier (green bar) prior to the phase set of V_m_ alternans. After that, the amplitude V_m_ and Ca^2+^ alternans gradually augmented.


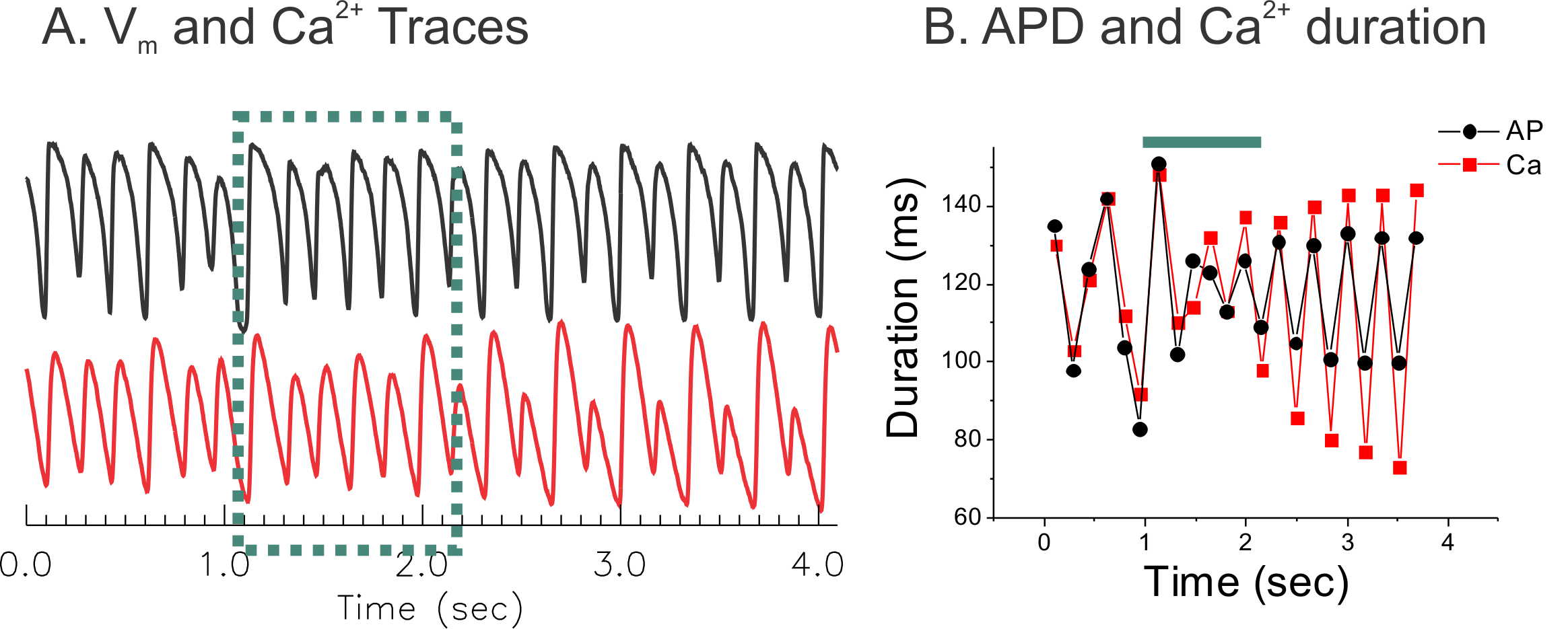


**S4 Fig. V_m_ and Ca^2+^ relationship during the phase transition of alternans.** (A) V_m_ (black) and Ca^2+^ traces (red). (B) APD (black circle) and Ca^2+^ (red square) duration plot. The green bar indicates the settling of new alternan phase.
