# Supplementary material for: Spatially Discordant Alternans and Arrhythmias in Tachypacing-Induced Cardiac Myopathy in Transgenic LQT1 Rabbits: The Importance of IKs and Ca2+ Cycling
Source: PLoS One. 2015 May 13;10(5):e0122754. doi: 10.1371/journal.pone.0122754 (PMC4430457; doi:10.1371/journal.pone.0122754)
Supplement: S5 File — (DOCX) [file pone.0122754.s005.docx]

### Discordant alternans in LMC-TICM

LMC-TICM also demonstrated discordant alternans. However, their dynamics were markedly different compared to LQT1-TICM. Figure S7 shows an example of discordant atlernans from LMC-TICM. The heart was stimulated from the left ventricle (panel A). The transition between odd and even beats was abrupt in Ca^2+^ in contrast to V_m_ (panel C), similar to LQT1-TICM. However, V_m_ and Ca^2+^ nodal lines (panel D) were quite stable without beat-to-beat changes. In addition, the patterns of V_m_ and Ca^2+^ nodal lines are very similar and Ca^2+^ alternans did not show complex phase transition unlike LQT1-TICM. This sharp contrast of discordant alternan behavior between LMC-TICM and LQT1-TICM indicates the important role of I_Ks_ as repolarization reserve to prevent complex repolarization dynamics and reduce induction of VF.


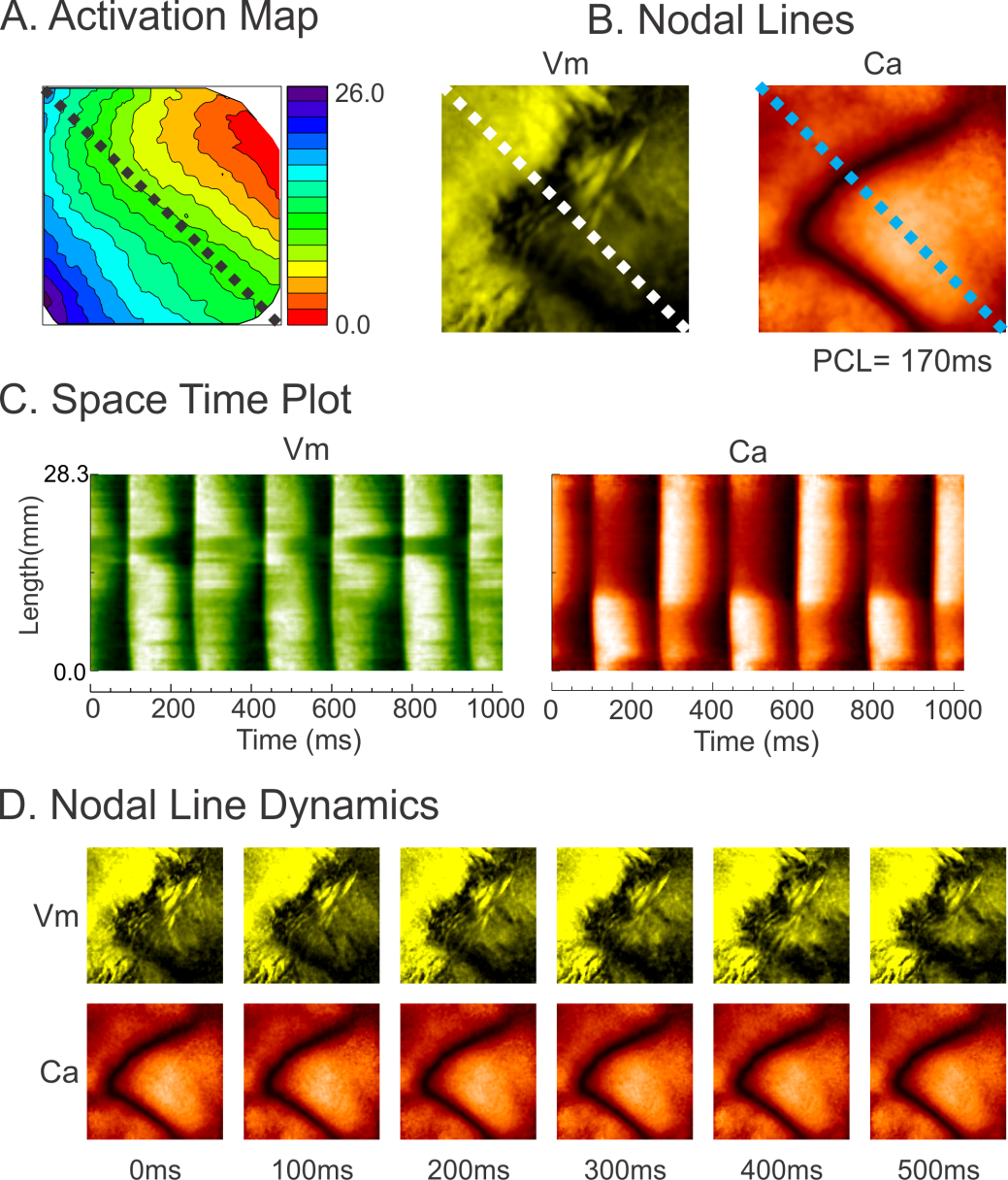


**S5 Fig. Discordant alternans in LMC-TICM.** A) Activation map. B) Nodal line images of V_m_ and Ca^2+^ at 170 ms pacing rate. The dark lines in the images mean small beat-to-beat changes in V_m_ and Ca^2+^ indicating nodal lines. C) Space-time plot of V_m_ and Ca^2+^ alternans along the dotted line in panel A and B. D) Series of V_m_ and Ca^2+^ nodal line images. LMC-TICM demonstrated relatively stable nodal lines associated with the conduction pattern.
